# The Hospital World

**Published:** 1910-05-07

**Authors:** 


### The Hospital World.

### BRITISH HOSPITALS ASSOCIATION.

By kind permission of the Treasurer and House Committee, a meeting of the Council of the above association was held at Guy's Hospital on April 28 at 4 p.m. Mr. H. Cosmo Bonsor was elected President for the year, and Mr. Conrad W. Thies Honorary Treasurer, while the following members were elected to the Council : Sir Henry Burdett, K.C.B., K.C.V.O., Sir E. Cooper Perry, M.D., Thomas Hayes, Esq., L. W. Glenton Kerr, Esq., J. D. Hedderwick, Esq., E. Stewart Johnson, Esq., Charles Lupton, Esq., Dr. Munro Moir, Henry Peterkin, Esq., J. A. Roxburgh, Esq., H. J. Collins, Esq., W. A. Tait, Esq. It was announced that Mr. A. William West had kindly consented to act as one of the Honorary Secretaries in conjunction with Dr. D. J. Mackintosh, M.V.O., and that Mr. A. F. Whinney, chartered accountant, of 32 Old Jewry, E.C., had kindly agreed to act as Honorary Auditor. The Treasurer's statement was submitted, and it was proposed that if arrangements can be made a conference shall be held in July in some large centre, probably Edinburgh, at which papers will be read and discussed. A vote of thanks to Mr. Cosmo Bonsor for presiding at the meeting was proposed and carried unanimously, after which the members present were taken round some of the wards and the nurses' home. The addresses of the honorary secretaries of the British Hospitals Association are as follows : Dr. D. J. Mackintosh, M.V.O., Western Infirmary, Glasgow, and A. William West, Esq., 3a Lyall Street, Belgrave Square, London, S.W.

### THE INCORPORATED HOSPITAL OFFICERS' ASSOCIATION.

By the invitation of the Manchester branch of this Association the members of the parent branch visited Manchester to inspect some of the hospitals of the city. Mr. Howell, Secretary of the Cancer Hospital, Fulham, President of the Association, was accompanied by about 30 secretaries and superintendents of London and Provincial hospitals. The visitors were welcomed by Major Tyler, Secretary of the Stockport Infirmary, President of the Manchester branch. The visitors, after calling upon the Lord Mayor of Manchester (Mr. Charles Behrens), who is a member of the Board of Management of the Manchester Royal Infirmary and Hon. Secretary of the Manchester Consumption Sanatorias, inspected the Children's Hospital, Out-Patient Department, Gartside Street, the Hospital for Skin Diseases, the new St. Mary's Hospital, Oxford Road (which is completed, but not yet occupied), and the Whitworth Street branch of the same institution. The Gartside Out-Patient Department was opened in 1907. At the time of the visit work was in full progress. The method of working the department was explained by Mr. H. J. Eason, Secretary of the Children's Hospital, Pendlebury. The Skin Hospital in Quay Street was next inspected, the Chairman (Mr. Sydney Hollins), the Secretary (Mr. Nall), and the Matron (Miss Boyd) explaining many matters of interest. The institution contains only 28 beds, but it has a most extensive out-patient department, and the equipment of the Finsen Light Department for the treatment of lupus, etc., is second only to that of the London Hospital. One half of the party paid a visit to the two branches of St. Mary's Hospital, one in Whitworth Street, the other an entirely new branch in Oxford Road, not yet occupied, and to which it is proposed to transfer women's and children's diseases, leaving the maternity work at the Whitworth Street branch. The two parties met again at the Midland Hotel, where they were entertained by the Manchester branch of the Association. After lunch both the London and Manchester members spent the whole afternoon at the Royal Infirmary. Mr. Carnt (General Superintendent), Miss Sparshott (Lady Superintendent of Nurses), Mrs. Morris (Lady Housekeeper), and Dr. Tylecote (Resident Medical Officer) acted as guides. After tea Major Tyler and Mr. Carnt expressed the pleasure the visit had given to the Manchester branch, and the President (Mr. Howell) returned thanks for the London Association for the hospitality extended to them. A few who were able to remain had the pleasure of visiting the Barnes Convalescent Hospital at Cheadle and the Manchester Children's Hospital at Pendlebury before returning to London the following day.

